# Accountability for large language models in health care

**DOI:** 10.2471/BLT.26.296186

**Published:** 2026-09-01

**Authors:** Carlos Fernando Mourão, Luiz Eduardo Juliasse

**Affiliations:** a Department of Basic and Clinical Translational Sciences, Tufts University School of Dental Medicine, One Kneeland Street, Boston, MA 02111, United States of America.

## Abstract

Large language models are entering clinical workflows faster than health-care institutions can assign responsibility for their failures. This situation is creating governance gaps with consequences that extend beyond individual patients to public health systems. The result is an accountability vacuum in which responsibility for harm mediated by large language models can be spread across clinicians, institutions and vendors. This responsibility gap distributes harm inequitably, particularly in low- and middle-income countries where regulatory infrastructure for digital health is still developing. We propose earned delegation as a predeployment standard: authority should be delegated only when evidence is proportionate to clinical risk, substantive human oversight is integrated and resourced, and responsibility for foreseeable failure modes is assigned in advance. To operationalize this standard, health systems should adopt a predeployment accountability charter specifying intended use, excluded use, validation evidence, oversight design, escalation pathways, auditability, subgroup performance review and named accountable parties. The charter should be integrated into existing institutional review, accreditation and procurement structures. Earned delegation provides a governance framework that health ministries, regulators and institutional leaders can adopt to ensure that use of large language models serves public health goals without outpacing them.

## Résumé

Les grands modèles de langage s’intègrent aux processus cliniques à un rythme plus rapide que celui auquel les établissements de santé parviennent à attribuer la responsabilité de leurs défaillances. Cette situation engendre des lacunes de gouvernance, dont les conséquences s’étendent au-delà des patients individuels pour affecter les systèmes de santé publique. Il en résulte un vide en matière de responsabilité, dans lequel la responsabilité des préjudices occasionnés par les grands modèles de langage peut être répartie entre les cliniciens, les établissements et les fournisseurs. Cet écart répartit les préjudices de manière inéquitable, en particulier dans les pays à revenus faibles et intermédiaires où le cadre réglementaire en matière de santé numérique en est à ses balbutiements. Nous proposons une délégation méritée comme norme préalable au déploiement: l’autorité ne devrait être déléguée que lorsque les preuves sont proportionnées au risque clinique, qu’une supervision humaine substantielle est intégrée et dotée des ressources nécessaires et que la responsabilité des modes de défaillance prévisibles est attribuée à l’avance. Pour mettre en œuvre cette norme, les systèmes de santé doivent adopter une charte de responsabilité préalable au déploiement précisant l’usage prévu, les utilisations exclues, les preuves de validation, la conception de la supervision, les procédures d’escalade, la capacité d’audit, l’évaluation des performances par sous-groupes et les parties responsables désignées. Cette charte doit s’intégrer aux structures existantes d’évaluation, d’accréditation et d’approvisionnement par des institutions. Cette délégation méritée offre un cadre de gouvernance que les ministères de la Santé, les autorités de régulation et les dirigeants d’établissements peuvent adopter afin de garantir que l’utilisation des grands modèles de langage erve les objectifs de santé publique sans les dépasser.

## Resumen

Los grandes modelos lingüísticos (Large Language Models, LLM) se están introduciendo en los flujos de trabajo clínicos antes de que a las instituciones sanitarias les dé tiempo a atribuir correctamente la responsabilidad por los errores derivados de su uso. Esta situación está generando brechas de gobernanza, cuyas consecuencias se extienden más allá de los pacientes individuales y afectan a los sistemas de salud pública. El resultado es una ausencia de rendición de cuentas, ya que la responsabilidad por el perjuicio causado por los grandes modelos lingüísticos puede repartirse entre los médicos, las instituciones y los proveedores de dichos modelos. Este vacío de responsabilidad distribuye el perjuicio de forma injusta, especialmente en aquellos países de renta baja y media en los que aún se está desarrollando la infraestructura reglamentaria en el ámbito de la salud digital. Proponemos la delegación basada en el mérito como norma previa al despliegue: la autoridad únicamente debería delegarse cuando el nivel de evidencia sea proporcional al riesgo clínico, cuando haya una supervisión humana real que esté integrada y dotada de recursos, y cuando se haya asignado con antelación la responsabilidad por los defectos previsibles. Para hacer operativa esta norma, los sistemas de salud deberían aprobar un documento de responsabilidad previa al despliegue en el que se especifique el uso previsto, el uso excluido, la evidencia de validación, el diseño de la supervisión, los procedimientos de escalamiento, la auditabilidad y la revisión del rendimiento del subgrupo, así como las partes designadas con obligación de rendir cuentas. Este documento debe integrarse en las estructuras ya existentes de revisión institucional, acreditación y contratación. La delegación basada en el mérito ofrece un marco de gobernanza que pueden adoptar los ministerios de sanidad, los organismos reguladores y las autoridades institucionales para garantizar que el uso de los grandes modelos lingüísticos esté al servicio de los objetivos de la salud pública sin rebasar su capacidad de control.

## ملخص

تدخل النماذج اللغوية الكبيرة في عمليات سير العمل الإكلينيكية بوتيرة أسرع من قدرة مؤسسات الرعاية الصحية على تحديد المسؤولية عن إخفاقاتها. يُؤدي هذا الموقف إلى ثغرات في الحوكمة، مع تبعات تتجاوز المرضى الأفراد لتشمل أنظمة الصحة العامة. والنتيجة هي عدم وجود مساءلة، حيث تتوزع مسؤولية الضرر الناجم عن النماذج اللغوية الكبيرة بين الأطباء والمؤسسات والموردين. تؤدي هذه الفجوة في المسؤولية إلى توزيع الضرر بشكل غير عادل، لا سيما في الدول ذات الدخل المنخفض والدخل المتوسط، حيث لا تزال البنية التحتية التنظيمية للصحة الرقمية قيد التطوير. نحن نقترح مبدأ التفويض المكتسب كمعيار قبل النشر: يجب تفويض السلطة فقط عندما تتناسب الأدلة مع المخاطر الإكلينيكية، وعندما يكون الإشراف البشري الفعال مدمجًا ومدعمًا بالموارد، وعندما يتم مسبقًا تحديد المسؤولية عن أنماط الفشل المتوقعة. لوضع هذا المعيار القياسي قيد التنفيذ، يجب على أنظمة الرعاية الصحية اتباع ميثاق للمساءلة قبل النشر، يُحدد الاستخدام المقصود، والاستخدامات المستبعدة، وأدلة التحقق، وتصميم الإشراف، وآليات التصعيد، وإمكانية التدقيق، ومراجعة أداء المجموعات الفرعية، والأطراف المسؤولة المُعينة. يجب دمج هذا الميثاق في الهياكل القائمة للمراجعة المؤسسية، والاعتماد، والمشتريات. يوفر التفويض المكتسب إطار عمل للحوكمة يمكن لوزارات الصحة والهيئات التنظيمية والقادة المؤسسيين اتباعه لضمان أن استخدام النماذج اللغوية الكبيرة يخدم أهداف الصحة العامة دون أن يتعداها.

## 摘要

大语言模型正在迅速进入临床工作流程，其速度远超过医疗保健机构界定失误责任的速度。这一情况形成了治理空白，其导致的后果已超出个体患者，延伸至公共卫生体系。其结果便是产生问责真空，由大语言模型介导产生的损害的责任分散至临床工作者、相关机构和供应商。这一责任缺口会导致损害后果分配不公，尤其是在数字医疗监管体系仍在建设之中的中低收入国家。我们提议将获权授权作为部署前标准：只有当证据与临床风险成比例、纳入实质性人工监督并配备相关资源，以及提前界定可预见的失误模式的责任，才可下放权限。为将这一标准付诸实践，医疗体系应当采纳一份部署前问责宪章，注明预期用途、禁用用途、验证证据、监督机制设计、逐级上报路径、可审计性、亚组性能评估以及具名责任方。此宪章应当被整合至现有的机构审查、资质认定以及采购体系。获权授权提供的治理框架可供各国卫生部门、监管机构及机构领导采用，以确保大语言模型的应用可为公共卫生目标提供服务，且不会超前于他们的节奏。

## Резюме

Большие языковые модели внедряются в клинические рабочие процессы быстрее, чем учреждения здравоохранения успевают определять ответственных за сбои в их работе. Такая ситуация приводит к кризису управления, что влечет за собой последствия не только для конкретных пациентов, но и для всей системы здравоохранения. В результате возникает вакуум подотчетности, при котором вина за вред, причиненный вследствие применения больших языковых моделей, может быть возложена одновременно на врачей, медицинские организации и поставщиков технологий. Этот дефицит ответственности приводит к тому, что бремя ущерба распределяется неравномерно, особенно в странах с низким и средним уровнем дохода, где нормативно-правовая база в сфере цифрового здравоохранения еще недостаточно развита. Мы предлагаем использовать принцип обоснованного делегирования в качестве обязательного стандарта, предшествующего внедрению. Это значит, что полномочия должны делегироваться только тогда, когда доказательная база соразмерна клиническому риску, контроль со стороны человека полностью интегрирован и подкреплен ресурсами, а ответственность за возможные сбои распределена заранее. Чтобы этот стандарт заработал на практике, системам здравоохранения необходимо перед использованием таких инструментов принять хартию ответственности, в которой прописаны целевое назначение технологии, запрещенные варианты использования, результаты валидации, структура надзора, алгоритмы эскалации, возможность проведения аудита, анализ эффективности в подгруппах и указаны поименно ответственные лица. Хартию необходимо интегрировать в уже существующие структуры институционального контроля, аккредитации и закупок. Принцип обоснованного делегирования предлагает структуру управления, которую министерства здравоохранения, регулирующие органы и руководители учреждений могут взять на вооружение, чтобы применение больших языковых моделей служило целям общественного здравоохранения и не выходило из-под контроля.

## Introduction

Consider a patient in a resource-constrained setting who receives a triage recommendation from a chatbot powered by a large language model, follows the recommendation and experiences harm after a delayed diagnosis. The clinician did not generate the recommendation; the health authority or institution approved the tool for a lower-risk purpose than the one for which it was actually used; and the vendor disclaims clinical liability. Each actor has a plausible defence, yet no party bears clearly assigned responsibility. This situation is the accountability vacuum: a structural gap in which harm mediated by large language models occurs without a predetermined responsible party. Rather than claiming that large language models create an entirely new problem, we argue that they intensify longstanding responsibility gaps in clinical decision support by combining broad task flexibility, persuasive natural language output, and fragmented lines of authority across vendors, health systems and end users. That combination now requires as much governance attention as technical performance before deployment of such a large language model. The consequences are not confined to individual clinical encounters: when accountability structures are absent, commercial large language model tools may be deployed across national health systems without the governance infrastructure needed to detect systematic failures, audit subgroup performance or assign responsibility at any level.

Three bodies of work affect this gap, and each stops short of closing it. Risk-tiered ethical frameworks match oversight to implementation risk but leave responsibility allocation to local discretion.^1^ Reporting guidelines, such as consolidated standards of reporting trials–artificial intelligence (CONSORT-AI); standards for reporting diagnostic accuracy–artificial intelligence (STARD-AI); transparent reporting of a multivariable prediction model for individual prognosis or diagnosis–large language model (TRIPOD-LLM); and chatbot assessment reporting tool (CHART), standardize how studies are described but do not bind institutions to act on what is reported.^2^^–^^5^ Regulatory instruments classify products by risk, govern market access and, in some jurisdictions, impose operational duties on deployers, but they stop at generic obligations rather than assigning ownership of specific clinical failure modes.^6^^–^^8^ The gap itself is not new: scholarship on automated systems has long described the responsibility gap that opens when an operator cannot foresee a learning system’s behaviour;^9^ the conditions under which meaningful human control preserves responsibility;^10^ and the tendency for the nearest human operator to absorb blame for failures of the wider system.^11^ What is missing for clinical large language models is a mechanism that converts these classifications and checklists into a named, enforceable chain of responsibility before they are used. That mechanism is what we set out to provide, using our expertise in translational clinical research and the ethics and governance of artificial intelligence (AI) in health care.

We aimed to define the ethical threshold for delegating clinical authority to a large language model, and to translate that threshold into an operational governance framework that specifies who is accountable, for what and before any harm. We state the standard (earned delegation), describe how the framework was developed, present the instrument (a predeployment accountability charter), and show its application through a worked case and a pathway for resource-constrained health systems.

## Development of the framework

We developed the framework through structured synthesis rather than formal consensus methods. We first collected the normative and empirical foundations for our argument from: (i) three regulatory or governance instruments (the European Union (EU) AI Act, the United States Food and Drug Administration (US FDA) clinical decision support framework, and the World Health Organization (WHO) guidance on AI and on large multimodal models);^6^^–^^8^^,^^12^ (ii) the leading risk-based ethical framework for implementation of clinical AI;^1^ and (iii) current empirical evidence on real-world performance of large language models and oversight for which we prioritized systematic reviews and randomized trials over examination-style benchmarks.^13^^–^^15^ We then derived the three conditions of earned delegation from recurring failure points in that evidence, and mapped each condition onto a corresponding, checkable charter element. To ensure that the charter contains verifiable requirements rather than general statements of intent, we aligned its data requirements with established reporting guidelines,^2^^–^^5^^,^^16^^–^^20^ so that each commitment corresponds to evidence a reviewer can request. 

### Earned delegation

In this framework, delegation concerns the sociotechnical configuration comprising the model, clinical workflow, human oversight and institutional controls, not the model as an autonomous moral agent. We propose earned delegation as the ethical threshold for use of clinical large language models. Delegation of clinical authority should not be presumed from fluency (natural and grammatically correct output of a large language model), benchmark performance (performance on standardized or simulated evaluation tasks) or commercial availability. Delegation should be earned through three conditions that must be satisfied before a large language model is permitted to influence care. First, the evidence must be proportionate to the clinical stakes, and evaluation should be made in the environment in which the large language model is intended to be used rather than relying exclusively on simulated or examination-style tasks. Second, human oversight must be substantive rather than nominal: clinicians must have the time, authority and workflow support needed to interrogate, reject or override model outputs. Third, responsibility for foreseeable failure modes must be assigned before a large language model is deployed to vendors, institutions and clinical services rather than negotiated after harm occurs.

This framework is compatible with risk-tiered approaches to clinical AI but adds a clearer requirement: evidence, oversight and accountability must be satisfied together, not treated as interchangeable substitutes. A system with promising performance but no credible oversight structure has not earned delegation. Nor has a system with nominal human review but no preassigned responsibility when a review fails. Researchers have argued that clinical AI should be evaluated according to the risks created by its implementation in practice, rather than solely by how readily its outputs can be explained,^1^ a position that supports earned delegation’s insistence on proportionate governance. When these conditions are not met, clinical authority is exercised through a sociotechnical configuration that has not earned it, and the accountability vacuum remains.

### Evidence for delegation

The current evidence remains too thin to justify broad clinical delegation. A recent systematic review identified 4609 peer-reviewed clinical studies on large language models published between January 2022 and September 2025. However, only 1048 studies used real-world patient data, of which just 19 were prospective randomized trials.^15^ Much of the literature still relies on simulated scenarios, examination-style tasks or limited samples. The Holistic Evaluation of Large Language Models for Medical Tasks (MedHELM) is an evaluation framework built on a clinician-validated taxonomy of five real-world clinical task categories (clinical decision support, note generation, patient communication, medical research and administration), which spans 22 subcategories and 121 tasks across 37 benchmarks and nine frontier models.^14^ We use this framework here because it directly measures the gap our paper is focused on. The framework shows that near-perfect performance on licensing-style examinations does not establish readiness for the heterogeneous, iterative and context-dependent work that dominates clinical practice, which is precisely the readiness that earned delegation requires evidence for.^14^

The common response is to assume that human oversight can compensate for these evidentiary gaps. That assumption is unsafe. A report shows that members of the public assisted by large language models identified correct medical conditions in fewer than 34.5% of responses in each group assisted by a large language model (600 responses per group), no better than controls using resources of their own choosing, despite the same large language models achieving 94.9% (1708/1800) accuracy when tested alone.^13^ Human review may fail not only because the model is wrong but because review becomes hurried or routine, or because reviewers give undue weight to apparently confident or authoritative model outputs. Oversight that exists on paper but not in practice does not mitigate delegation risk; it merely obscures where responsibility will fall once harm occurs.

### Risks at the patient interface

This responsibility gap is most pronounced at the patient interface, where the communicative strengths of large language models can paradoxically undermine the autonomy they are intended to support. A recent paper argues that large language models could serve as epistemic scaffolding for informed consent by facilitating iterative questioning and clarifying patient values.^21^ While this potential exists, a confident tone, systematic presentation and apparent comprehensiveness can amplify framing effects, whereby decisions are influenced by how information is ordered, emphasized or presented. When a large language model sequences consent information or defaults to particular treatment framings, it decides what appears relevant, decisions traditionally subject to clinical accountability. In the absence of predetermined governance, outputs are trusted for their authoritative tone rather than their accuracy, leaving patients unable to distinguish between the two. Where large language models are used to facilitate the consent process, the accountability charter should require disclosure to patients that AI is shaping the information structure, and should designate responsibility when framing effects contribute to suboptimal decisions.

## Accountability charter

Addressing the accountability gap requires a concrete governance mechanism: health systems using clinical large language models should adopt a predeployment accountability charter specifying, for each use case, which party bears responsibility at each decision point. To ensure enforceability, this charter should be integrated into existing institutional oversight, accreditation processes and procurement requirements. Risk stratification provides the organizing structure. For low-risk administrative tasks, lighter oversight may be sufficient. For high-risk functions such as triage, diagnostic reasoning, treatment recommendation and consent facilitation, the charter should mandate prospective local validation, version-locked audit trails, defined override protocols and named responsible parties for each failure mode (Box 1).

Box 1 **Minimum content of a predeployment accountability charter for clinical large language models**

- Intended use and excluded use
- Risk tier and justification
- Validation evidence available before deployment and revalidation triggered on any model or version change
- Named accountable parties at vendor, institution and service-line level
- Human oversight design, escalation rules and oversight-integrity monitoring, including override rates, review time, escalation frequency and unexplained drift towards near-total concordance with model recommendations
- Equity audit, including subgroup performance reporting
- Audit trail, incident reporting and review schedule
- Conditions for suspension or withdrawal

Specifying who is accountable to whom is the core work of the charter and it falls across four roles. The vendor is accountable to the institution for model performance, version integrity and disclosure of known limitations, judged against reporting-guideline items.^2^^–^^5^^,^^16^^–^^20^ Because hosted models can change without notice, the charter also requires contractual notification of any model or version change, with revalidation before continued clinical use. The institutional governance body, typically the entity that already runs clinical, ethics and procurement reviews, is accountable to patients and the health authority for approving the intended use, the risk tier and the oversight design, and for suspending the tool when thresholds are breached. The clinical service line is accountable for operating the tool within its intended use, exercising override and reporting incidents. The national regulator or the health ministry sets minimum governance requirements, which institutions may strengthen according to local risk, and adjudicates disputes. Each charter names a specific accountable party at the vendor, institutional and service-line level for every foreseeable failure mode, so that when review fails, responsibility is located rather than negotiated.

Several reporting standards support the rigour this charter demands. STARD-AI provides a checklist for comprehensive and transparent reporting of AI-centred diagnostic accuracy studies, enabling assessment of how AI systems are evaluated.^3^ TRIPOD-LLM requires transparency on model versions, prompts, deployment context and human oversight provisions.^4^ CONSORT-AI provides the reporting standard for clinical trials of AI interventions,^2^ while CHART provides 12 items and 39 subitems for reporting studies of chatbots that summarize clinical evidence and give health advice.^5^ A broader system of complementary guidelines, including developmental and exploratory clinical investigations of decision support systems driven by artificial intelligence (DECIDE-AI);^20^ generative artificial intelligence tools in medical research (GAMER);^17^ prediction model risk of bias assessment tool (PROBAST)+AI;^19^ reporting checklist for foundation and large language models (REFINE);^18^ and TRIPOD+AI,^16^ further supports rigorous predeployment evaluation. The accountability charter operationalizes these frameworks by converting reporting requirements into binding institutional commitments.

### Applying the charter

To show how the framework operates in practice, we return to the opening scenario and specify what the charter that earned delegation would have required before the triage chatbot went live. The example is illustrative and created to demonstrate application; it is not a report of a system that has already been deployed and no such prospective institutional validation has yet been conducted. A district hospital proposes to deploy a symptom-triage chatbot powered by a large language model at first contact. Because triage affects decisions about the urgency and level of care a patient receives, the tool is classified as high-risk, which triggers the full charter (Box 2); where the EU Artificial Intelligence Act applies, a system of this kind may fall within Annex III of the Act, so the classification follows from law rather than local judgement alone. The distinguishing feature is not that the tool is proven safe, but that every foreseeable failure mode has a named owner and a checkable safeguard before any patient is exposed. Under-triage (where a patient’s health problem is initially assessed as less severe than it actually is), is measured prospectively on local presentations rather than inferred from examination-style accuracy, low-confidence outputs are escalated automatically to a named clinician, and performance is reviewed monthly, disaggregated by age, sex and language, against pre-set thresholds for suspending the chatbot. In the opening scenario, this charter would have prevented the tool from being used beyond its approved purpose and would have located responsibility rather than leaving each actor with a plausible defence.

Box 2 **Illustrative application of the predeployment accountability charter to a high-risk symptom-triage chatbot**

**Charter element and specification for the triage chatbot**

**Intended and excluded use**

Symptom-based triage guidance for adults. It is explicitly excluded from paediatric triage, mental-health crisis and any use as a substitute for clinician assessment.

**Risk tier**

High-risk, because triage affects decisions about urgency or level of care and hence has the potential to cause harm if triage decisions are incorrect. The full charter is required.

**Validation before deployment**

Prospective evaluation on local patient presentations before going live, with the chatbot operating in the background and its outputs not used for patient care; under-triage rate is used as the safety-critical metric rather than examination-style accuracy. Revalidation is required before continued use after any model or version change.

**Named accountable parties**

- Vendor: designated clinical safety officer for model performance, version integrity and notification of any model or version update
- Institution: deputy medical director as charter owner responsible for approval of intended use, risk tier and oversight design
- Service line: head of emergency services, responsible for real-time override and incident reporting.

**Oversight and escalation**

Clinician confirmation required before any decision about urgency or level of care. Low-confidence or red-flag outputs are automatically escalated to a named on-call clinician. Monthly review of override rate, time-to-decision and concordance drift are oversight-integrity metrics.

**Equity audit**

Monthly under-triage review disaggregated by age, sex and language of interaction required.

**Audit trail and review**

Version-locked logging of every input, output and override. Monthly safety review required against preset suspension thresholds, with quarterly independent audit of the log.

**Suspension and withdrawal**

Prespecified triggers are under-triage rate above the locally agreed threshold on two consecutive monthly reviews, or any single sentinel under-triage event. The named institutional clinical lead may suspend use immediately and must do so within 1 working day of a confirmed breach; reinstatement requires revalidation and documented approval by the governance body.

Note: The example is constructed to demonstrate how earned delegation would be operationalized before deployment; it is not a report of an implemented system. For each charter element, the table specifies who is accountable and which safeguard applies to the corresponding failure mode.

### Bridging the implementation gap

The proposed charter complements existing regulatory systems while addressing their gaps. The US FDA’s evolving framework for clinical decision support software classifies AI tools by risk but does not recommend how institutions should allocate accountability for specific deployment failure modes. ^8^ The EU Artificial Intelligence Act classifies clinical AI as high-risk by two routes: (i) as a safety component of or as a regulated medical device requiring third-party conformity assessment; and (ii) through the use of cases listed in Annex III of the Act, which directly names emergency triage systems for patient health care. In Article 26, the Act imposes operational duties on deployers, including named human oversight by competent natural persons (that is, humans), monitoring, log retention and disclosure to individuals subject to AI-assisted decisions. Article 27 requires a fundamental rights impact assessment of public bodies and private providers of public services. These duties are generic and system-level rather than specific to a case. They do not require a health system to identify, before deployment, which party owns each foreseeable failure mode of a particular clinical application. Furthermore, the duties are binding only within the EU, leaving most of the world’s health systems outside their reach.^6^ The WHO guidance on ethics and governance of AI for health provides core principles, including transparency, accountability and equity, but does not mandate binding predeployment commitments at the institutional level.^7^ The updated WHO guidance on large multimodal models reinforces these principles while acknowledging that rapid commercial adoption of large language models has outpaced governance capacity, particularly in low- and middle-income settings.^12^ The accountability charter bridges this implementation gap by translating regulatory classification, deployer duties and ethical principles into institutional obligations tailored to each clinical application, specifying who is responsible, for what and before any harm occurs, and by extending the same requirements to jurisdictions with no equivalent statutory system. For health ministries and national regulators, the charter provides a mechanism that can be adapted to local legal and institutional contexts while maintaining consistent governance across different health systems.

### Equity and implementation

Justice necessitates this governance structure because the current accountability failure distributes harm inequitably both within and between health systems. Performance may not have been evaluated separately across relevant patient groups. Commercial incentives may influence outputs without transparency. Patient data may be repurposed without governance. The resulting harms disproportionately affect people with the least institutional power to identify errors, contest decisions or seek redress. This inequity is most acute in low- and middle-income countries, where digital health regulatory infrastructure is often underdeveloped, health workforce shortages may increase reliance on automated tools, and commercial vendors may deploy large language models with even less oversight than in settings with more resources.

Because a full charter can be resource-intensive, we propose a phased pathway so that resource-constrained systems can adopt the basic components first and add depth over time. Phase 1 (documentation) requires only intended-use and excluded-use statements, a risk tier and a named accountable party. These low-cost commitments already prevent the most common failure, namely, use beyond approved purpose. Phase 2 (basic safeguards) adds a defined override step and disaggregated reporting of one safety-critical metric, such as under-triage rate by subgroup. Phase 3 (full auditability) adds version-locked logging and scheduled independent review as the capacity allows. Regional or national bodies can host a shared charter template and pooled audit function so that individual facilities are not each rebuilding governance from scratch. This phased model lets health ministries have a consistent accountability level while scaling the more demanding audit procedures to local capacity.

The accountability charter must therefore include equity auditing provisions, such as mandatory disaggregated performance reporting by demographic subgroup and transparent documentation of training data composition. Guidelines such as PROBAST+AI^19^ and TRIPOD+AI^16^ further enable this accountability by explicitly calling for disaggregated performance reporting by demographic and clinical subgroups. Without such provisions, the use of large language models risks reproducing and amplifying the health disparities that public health governance exists to reduce.

### Importance of governance context

Emerging evidence from trials supports the principle that implementation context matters for the outcomes of the use of large language models. In a randomized controlled trial with 111 specialists across 24 medical disciplines at two health centres, with 2069 patients randomized, the authors evaluated a large language model chatbot co-designed with local stakeholders for transitions from primary care to specialist care.^22^ Relative to the no-chatbot arm, the co-designed system reduced mean physician consultation duration from 4.41 minutes (standard deviation, SD: 2.77) to 3.14 minutes (SD: 2.25), a reported reduction of 28.7%, and improved patient-reported ease of communication. Two findings relate to implementation and development design rather than model capability alone: outcomes were equivalent whether patients used the system independently or with staff support, and, in a separate pretrial simulation, the co-designed system received higher expert ratings than a version of the same base model additionally fine-tuned on local primary-care dialogues. This nonrandomized comparison cannot attribute the difference to any individual design component, but it indicates that co-design and deployment structures may add value beyond local fine-tuning alone. While this trial did not test the accountability charter framework proposed here, it suggests that co-design and implementation context can influence the clinical use of large language model systems, which reinforces the argument that the context in which a large language model is deployed deserves attention alongside model performance.

### Limitations

Our proposed charter has limitations. The framework was developed through structured conceptual synthesis rather than a formal consensus process and has not yet undergone prospective implementation testing. Patients, community representatives, health ministries and frontline professionals from low- and middle-income settings were not involved in its initial development, and the resource requirements of the phased pathway have not been costed. Participatory adaptation and prospective evaluation across different health systems are therefore needed before the charter can be considered validated or broadly generalizable.

## Conclusion

Health-care systems should address the accountability gap before deployment of large language models becomes difficult to govern consistently across settings. Large language models are ethically defensible when their authority is confined, their limitations are transparent and they operate within governance structures that assign responsibility in advance. The ethical boundary is not defined by imperfection, as human systems are also imperfect. Rather, the boundary is reached when an imperfect model assumes the clinical authority it has not earned within a system that has not determined who is accountable when harm occurs. Earned delegation and the predeployment accountability charter give health ministries, regulators and institutional leaders a concrete, adaptable instrument to close the accountability gap. The immediate next step is prospective evaluation of the charter in real deployment situations, including in low- and middle-income settings.
